# Digestion, Exercise & Sleep

**Published:** 1874-07

**Authors:** W. C. Coleman


### DIGESTION AS INFLUENCED BY EXERCISE AND SLEEP.

W. C. COLEMAN, M. D.

An eminent Medical Professor* has said: "The value of preventive medicine is the greatest of all questions connected with the science and art of medicine."

In America it is generally believed, and almost universally conceded, that diseases of the nutrient system are the most prevalent, and especially those appertaining to digestive apparatus. Therefore any information on this subject which would in any degree assist in preventing such diseases will doubtless be gladly received by the public, and especially all those who in any way may have derangements of the nutrient system. It will be our aim to base our statements on well established facts as derived from actual observation and experiment and in accordance with physiological principles. Digestion is the reduction of food to a soluable mass and its convertion into a form in which it may be taken into the blood, and from thence be received and appropriated to the various tissues of the body. The food is rendered soluble in the stomach, and some articles are entirely digested in this portion of the alimentary canal and absorbed without coming in contact with any digestive fluid save the gastric juice, while other articles after they have been rendered fluid in the stomach, immediately pass on to the small intestines, where they mingle with the other fluids, and digestion is completed. The stomach has muscular tissue entering into its formation, and like other portions of the muscular system, has the power of motion, and by the action of the muscular walls of this organ the food during healthful digestion is kept in constant motion.

The process of digestion may be greatly hastened by engaging in light exercise, either physical or mental, as pleasant company and cheerful conversation, without argument, or engaging the mind lightly in reading the current news, or gently walking to and fro viewing the beauties of nature or art, or in some similar exercise for a short time after meals. The time required to digest an ordinary meal of food, properly prepared and well masticated, varies from two to four hours. The average time may therefore be placed at three hours, in which all solid food is rendered fluid, the chemical changes which necessarily precede absorption are fully accomplished. During the time the stomach is reducing the food to fluidity, its work is much more laborious than afterwards, and it is at this period that other portions of the body should not be called into active exertion, and as a rule it may be said that in less than one hour the work of separation and rendering fluid is well advanced in healthful digestion, while the entire process may not be completed for a considerable length of time afterwards. The gentle mental exercise derived from leisurely looking over a paper, magazine or book, or the emotional sensations of a pleasant chat, or the enlivening feelings experienced in viewing nature or art beauties, have such a mildly stimulating and tonic influence on the nervous system and digestive organs, that experience and custom have given them high rank as aids to digestion without referring to either physiology or hygiene.

Anger, grief or great mental excitement of any kind has directly the opposite effect and thus digestion is greatly impeded. There are few who have not seen a "fit of indigestion" produced by undue mental excitement, and almost every one knows how suddenly the appetite disappears when distressing or alarming news are received just at meal time, and if food has been taken, how that indescribable feeling which medical writers call malaise, immediately makes its appearance. It is, therefore, very evident that the mind should not be called into active or prolonged exertion immediately after meals, if we would avoid impeding digestion. That violent physical exertion is equally injurious is readily proved by actual experiment. It is impossible to put forth great effort physically immediately after a full meal, without experiencing unpleasant sensations in the region of the stomach, which will be followed by feelings of exhaustion, and a continuation of such experiences rapidly proves injurious to the general health, and much more food is required to sustain the

*Da Costa.

body than when the exercise is gentle and unexciting after meals.

Every good horseman has learned by experience that his horse will not endure great labor and tires readily if driven rapidly or drawn heavily just after feeding, and that much more food is required to keep him in a good condition. These observations of the horsemen are in strict accordance with physiological principles and are no less true regarding man than animals, and are just as susceptible of practical application in the one case as the other. It is only necessary that man be consistent and treat himself as kindly as he does his favored horse, and simply give the digestive organs a chance to perform their work in a proper manner, and he will find that he can perform a greater amount of labor in a given length of time and with greater ease, and maintain his physical powers in a more vigorous condition.

It is a fixed law in the human economy that every portion of the body should have its appropriate share of blood, and during the period of digestion the vital energies are to a great extent directed to that function, and the digestive organs require more blood than at other times. Therefore, if the muscular system be called into violent action immediately after food is taken, blood must be detracted from the stomach, and should the mind be called into active employment or deep study, the brain which is the material object through which the mind acts must have a large supply of blood, and thus the process of preparing the food for nourishing the tissues will be very imperfectly performed, and both the physical and mental labor will be accomplished with great difficulty and very generally in an imperfect manner.

*In adult life food is imperfectly digested during sleep.*

This is no unfounded theory, but the numerous observations made on St. Martin, the greatest of living wonders, have proved beyond a doubt that the natural condition of stomach during sleep is quiescent and collapsed, and when this condition is disturbed by recently ingested food, the sleep will be unsound and much less refreshing and invigorating than when the process is fully completed before the hours of repose, and the food will be digested with great difficulty and almost invariably its nutrient properties will not be fully prepared to enrich the blood. The experience of all classes fully confirms this statement, and yet there are a few who deny its truthfulness and claim that sleep is in no way influenced or affected by digestion, and attempt to prove their theory by their own personal experience. Among intelligent people such persons are extremely few. There are, however, not a few among the more opulent who disregard the teachings of their own experience and violate this natural law by eating great suppers late at night—not regularly, but occasionally—and still many of such enjoy a fair average of health. It will be found by observation that most of those who thus indulge and are not perceptibly injured, have learned to carefully observe a great majority of the more common laws of life and health, and that over indulgence of the appetites requires rest, and thus by adhering to a well established principle in hygiene, "much exhaustion requires much rest," they permit their over-taxed digestive organs to recuperate previous to calling them into any active exertion. This proves nothing in favor of late suppers, but very clearly illustrates *what a man may endure and not what he might enjoy.*

But what of the man who invariably takes the fourth meal and then retires for the night and claims that he enjoys "excellent health." It is only bodily health this man possesses, not a free, clear and active mind, for be it remembered he is not a *great thinker*, notwithstanding he is a *great eater.* He is the muscular man, the full blooded fellow, with strong bones, whose brain is unaccustomed to mental activity and is not susceptable of vivid mental impressions.

He is in truth and reality an imitator and not a thinker, and his bodily health is secured by his physical exertions, fresh air and other favorable hygienic circumstances with perhaps a good natural constitution inherited from vigorous parents. But take the history of these exceptions, and we almost invariably find that their latter days are full of sorrow, and they lament thus: "Formerly I could eat all kinds of diet, and always felt well, but now I cannot eat even the plainest articles and feel comfortable." Previous to the decline of life the vital powers may resist successfully the influence of over-taxed digestive organs and a want of sufficient sleep, but in after life, the effects will be so destructive of nutrition that the general health must sooner or later become disordered. This is fully in accordance with physiological principles, and when we contemplate the matter in that light, we learn that the nervous system is made up of the brain, the spinal cord and nerves, and that this system *presides over all the motions of the body, both voluntary and involuntary, as well as the sensations.* It is a well-known fact, the brain is the centre of the nervous system, or is the great nerve center, and is the material object by or through which the mind acts, and thus the voluntary movements are directed by the will.

The involuntary movements are directed and controlled by and through the same portion of the physical being, and when the nervous system is injured, diseased, or its influence withdrawn from any portion of the muscular system, there will be a corresponding derangement of the motions and functions of that part, as is well illustrated in paralysis, St. Vitus dance, or tetanus.

The heart is a muscular organ and its motions are strictly involuntary, and the lungs are enveloped in a muscular and bony case, which acts to a great extent involuntary, and we therefore may take the movements of the heart and lungs as true types of *involuntary motion.* The stomach having its muscular walls, has also motion, which is involuntary but not constant like those of the heart and lungs, only periodical, and therefore we may say the motion of the stomach during digestion is a true type of *periodical involuntary motion,* while those of respiration and circulation are true types of *constant involuntary motion.* We are entirely insensible of these operations unless our attention be especially invited to them, and yet, notwithstanding this, the blood is being driven with great rapidity through all the tissues, and vast quantities of air are absorbed and exhaled, and during the period of digestion the food is kept in constant motion and chemically changed to enrich the "vital fluid" for the nourishment of the various tissues ; but suppose there is great muscular effort put forth immediately after a full meal, or there be any sudden emotional excitement, such as fright, or grief, and the result is a disturbed action of both the lungs and heart, and the process of digestion is arrested at once, which will be followed by feelings of general depression and derangement of health. The work of the nervous system during sleep is to preside over the constant involuntary movements of the body—circulation and respiration being the principal, and therefore any additional exertion must naturally result in exhaustion. The great work of nourishing the tissues is especially favored during sleep, and must *follow digestion* from the very fact that the tissues cannot be nourished until the nutriment contained in the food is received into the blood, and this can not take place previous to digestion. Therefore if the nervous system—which should be devoted to the work of respiration and circulation during sleep—is called to preside over the process of digestion and cause an increase flow of blood to the stomach, and thus the nervous system overworked, which must be followed by exhaustion if presisted in, and no man who makes proper use of his brain during the wakeful hours can thus abuse it during sleep without injury, and his mind will be aroused and the repose disturbed by unpleasant and frightful dreams.

It may be asked, why do infants sleep soundly immediately after taking food. The nervous system of the infant is not required to superintend any great physical exertion, and the mental operations extend only to the immediate wants of the physical being, hence the work of the brain is almost wholly devoted to the work of nutrition, there being but little effort in search of knowledge, and but slight voluntary exertion, except perhaps the *occasional cultivation of the voice early in the morning.*

Therefore the brain of the infant is performing its natural functions during sleep while it presides over *digestion* as well as respiration and circulation, and the growth and nourishment of the tissues are especially favored during sleep, and thus it is that the physical organization continues to be developed from birth, while the mind is comparatively inactive and uncultivated for a considerable length of time, and it is only when the child has begun to use his mental powers, and take more solid food that we find sleep disturbed by digestion, unless it be from real disease or over indulgence of the appetites.

The same is true of adults who have been sick and confined to bed for a time, their food being plain and of easy digestion, and if the brain has been free from disease and the mind undisturbed from any cause, there may be refreshing sleep during the period of digestion, but this, like all other exceptions to the general rule, only prove the more conclusively that in health digestion should be completed before the hours for sleep.

